# The Hot Phonon Bottleneck Effect in Metal Halide Perovskites

**DOI:** 10.1021/acs.jpclett.4c03133

**Published:** 2024-12-16

**Authors:** T. Faber, L. Filipovic, L.J.A. Koster

**Affiliations:** † Zernike Institute for Advanced Materials, University of Groningen, Nijenborgh 4, 9747 AG Groningen, The Netherlands; ‡ CDL for Multi-Scale Process Modeling of Semiconductor Devices and Sensors at the Institute for Microelectronics, TU Wien, Gusshausstrasse 27-29, 1040 Vienna, Austria

## Abstract

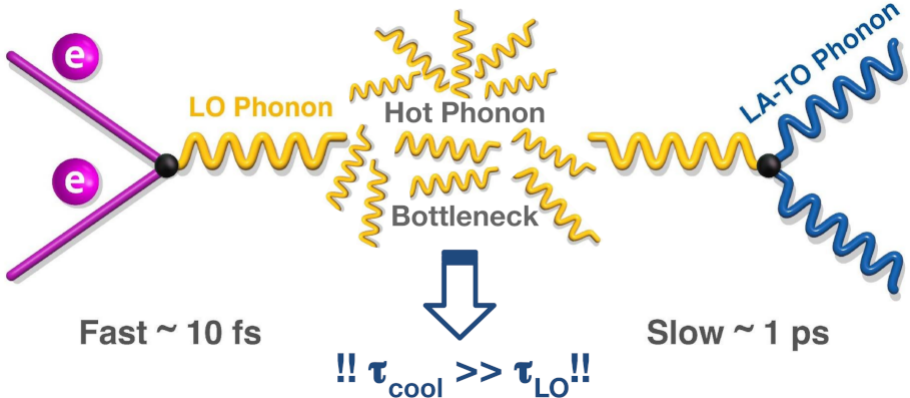

The hot phonon bottleneck (HPB) effect has been proposed as one of the main phenomena behind the slow cooling in metal halide perovskites. Even though consensus has been reached regarding its existence, open questions remain concerning the HPB’s specific applicability and potential regarding hot carrier solar cell (HCSC) applications. We present a full investigation using ensemble Monte Carlo simulations of the HPB effect in metal halide perovskites (MHP). After describing the HPB effect in detail, we quantify how the HPB effect can extend carrier cooling times by orders of magnitude. We show how the HPB effect depends on carrier concentration, longitudinal optical (LO) phonon lifetime, and LO phonon frequency and connect these findings to how MHPs should be tuned concretely. Using ensemble Monte Carlo simulations, we can accurately model the interplay between carrier–phonon and carrier–carrier interactions up to high carrier density, yielding precise predictions regarding the HPB effect. This study provides important insights into the governing dynamics behind the HPB effect and shows how cooling times can be extended far beyond the phonon lifetime. Furthermore, it contributes to the discussion on cooling times in MHPs and their suitability for HCSC applications.

Metal halide perovskites (MHP) have had the center stage of photovoltaic research, since the moment they were first introduced.^1,2^ Due to their remarkable optoelectronic properties and defect tolerance, power conversion efficiencies of 26% have been reported.^3^ Additionally, MHPs have been a promising candidate for hot carrier solar cell (HCSC) applications, as numerous experimental reports have been published on slow hot carrier cooling.^4^ Slow carrier cooling is the crucial component for a material to qualify as an active layer in a HCSC.^5^ As was recently reviewed by Fu et al., a plethora of studies was published on the underlying intrinsic photophysics, considering several possible mechanisms such as the hot phonon bottleneck effect,^6−16^ polaron screening,^17−21^ Auger heating,^7,14,15,22,23^ band-filling,^24,25^ diminished electron–phonon coupling,^26^ and an intrinsic phonon bottleneck effect.^27,28^

Among these, the hot phonon bottleneck (HPB) effect is most widely accepted for its role in the extended cooling times.^4^ Generally, the physical picture behind the HPB effect is well understood for polar semiconductors.^29−34^ It was first reported for MHPs by Yang et al. in 2016.^6^ The HPB effect arises from a nonequilibrium population of longitudinal optical (LO) phonons,^35^ and can be best understood when considering the full energy relaxation diagram:^36^

1. carrier → LO phonon. As MHPs are polar crystals, carriers interact strongly via LO-phonon coupling mediated via Coulomb interaction.^37,38^
2. LO-phonon → acoustic phonons. For cubic structures, this interaction takes place predominantly via the symmetric Klemens^39^ decay (LO → 2 LA phonons, and subdominantly via the antisymmetric Ridley^40^ decay (1 LO → 1 LA + 1 TO phonon).^41^ LA and TO refer to longitudinal acoustic and transverse optical, respectively.
3. acoustic phonons → thermal reservoir. Acoustic phonons conduct the energy in the form of heat away to the far-field region in the material.

As the LO-phonons are mainly produced around the zone center (stage 1), they have a rather low dispersion, causing them to largely remain in the photoexcited volume.^42^ If, at high excitation densities (ρ > 10^18^ cm^–3^), the carrier–LO phonon scattering rate (stage 1) is much higher than the LO phonon decay rate (stage 2), a nonequilibrium LO-phonon population can build up.^43^

The HPB effect is a resultant effect arising from the discrepancy in decay times between carrier–phonon and phonon–phonon interactions. In Figure 1, a visualization of the effect is presented. The nonequilibrium LO phonon population, arising from the discrepancy, results in an increased probability of reabsorption by charge carriers. This is essentially how the HPB effect extends carrier lifetimes. LO phonons are swiftly reabsorbed, keeping carriers hot, instead of decaying further and converting carrier energy into heat. The HPB effect thus arises from the complex competitive interplay between both the carrier–phonon and phonon–phonon interactions, and it is not only the phonon lifetime that determines the scale of the HPB effect.^7^ Therefore, it is not clear to what extend the HPB effect can elongate cooling times.

**Figure 1:**
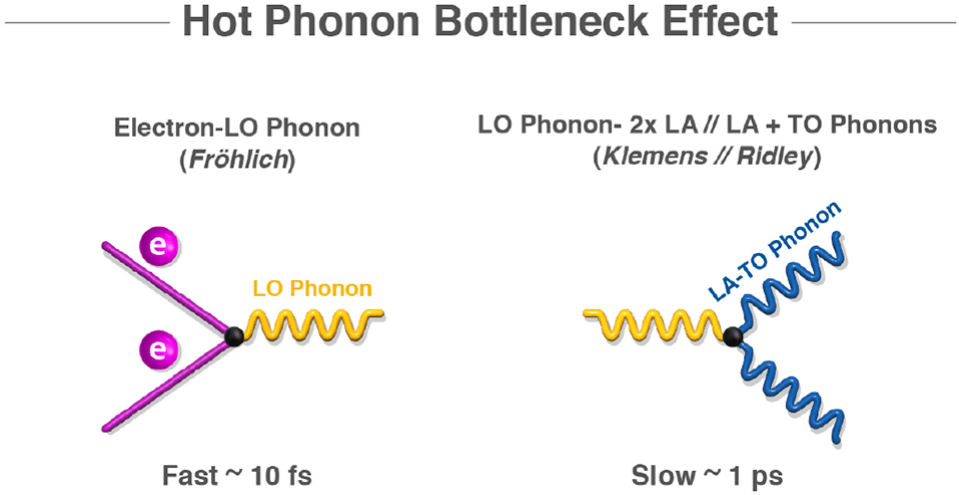
HPB effect arises due to a fast electron–phonon coupling rate, and a relatively slow phonon decay rate. In MHPs, the former is governed by the Fröhlich interaction, while the latter by the Klemens or Ridley decay.^39,40^

In this study, we show how the HPB effect can extend carrier cooling times far beyond LO phonon lifetime for metal halide perovskites, by observing the impact of the carrier concentration, LO-phonon lifetime, and LO phonon frequency. To model the particle dynamics through the entire cooling process, we use ensemble Monte Carlo (EMC) simulations.^44−48^ When dealing with nonequilibrium charge transport, the EMC method is a widely recognized numerical method to solve the Boltzmann transport equation.^49^ In a previous study, we showed, using EMC, what the role is of thermalisation on the cooling dynamics of hot carriers in MHPs.^50^

Our methodology allows us to accurately model the interplay between carrier–phonon and carrier–carrier interactions up to high carrier density, yielding precise predictions regarding the implications of the HPB effect. Moreover, by using ensemble Monte Carlo simulations, we are able to directly visualize our findings in a clear and concise way. For modeling the HPB effect, we use the algorithm developed by Lugli et al.^51^ It has been used together with the EMC for GaAs,^52,53^ for quantum wells in GaAs,^54^ or InGaAs/InGaAsP;^55^ however, it has never been applied in the context of perovskites.

Our model considers a single-mode approximation of the Fröhlich interaction. Even though this approximation holds well for certain types of perovskites,^56^ it has been shown for MAPbI_3_ that the consideration of multiple phonon modes is important in explaining transport properties such as the temperature dependence of the mobility.^57,58^ To solve this, one can consider an effective phonon mode coupling, composed of a mixture of multiple modes and resonating at a resultant frequency.^59−62^ However, the fundamental nature of the Fröhlich interaction that leads to the HPB effect is not altered by multiple phonon modes even though the coupling amplitude will be different. More important is the approximation regarding the weakly dispersive nature of the phonons, which in perovskites is shown to hold well.^63^

As our findings relate to preferable concrete chemical compositions, we can formulate predictions on how perovskites should be tuned in order for them to maximally harness the full potential of the HPB effect. Our results show how extended cooling times can be achieved far beyond the LO phonon lifetime, and give important insights into the governing dynamics behind the HPB in MHPs.

Our simulations were performed with the ensemble Monte Carlo code ViennaEMC,^64^ consisting of carrier–LO-phonon interactions, and carrier–carrier scattering. We have integrated a fast multipole method (FMM) to compute the carrier–carrier interactions, for which we used the ScalFMM package.^65^ For a more extensive overview on the ensemble Monte Carlo method we refer the reader to the Supporting Information.

The transition rate for the carrier–LO-phonon or Fröhlich interaction is given by^47^
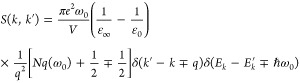
1where *e* is the elementary charge, ω_0_ is the typical phonon frequency, *V* is the volume, and ε_*∞*_ and ε_0_ are the optical and static parts of the dielectric constant, respectively. The phonon occupation number is given by *N*_*q*_ for absorption (−) and *N*_*q*_ + 1 for emission (+).

The LO-phonon scattering rate for both absorption and emission is computed by performing an integration over *q*, or all final states. One obtains:^44^
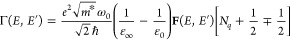
2where m* is the effective mass, *E* is the energy of the carrier, and **F**(*E*, *E*′) is a function of the energy *E* before, and after the *E*′ interaction with a phonon, where *E*′ = *E* ± *ℏω*_0_ for absorption (−) and emission (+), respectively.

The phonon occupation number *N*_*q*_ determines how many phonons occupy that specific mode. This is the quantity of interest for this paper, as it essentially determines the HPB effect. An increase in *N*_*q*_ will alter the emission and absorption rates, bringing them closer together, as the relative factor of  becomes less and less dominant for larger values of *N*_*q*_. If the LO emission and absorption rates come closer together, carriers cannot lose their energy accordingly, hence resulting in longer cooling times. This is essentially how the HPB effect translates a nonequilibrium population of LO-phonons into extended carrier cooling times.

In our previous paper, we defined a perovskite-like system by its effective mass m*, dielectric constants ε_0_ and ε_*∞*_, and the typical phonon frequency ω_0_.^50^ Our initial conditions consist of a perovskite-like system in thermal equilibrium at room temperature. Our simulations start with an energy pulse of 0.5 eV to model the photoexcitation by a laser. We track all the particle momenta and positions over time, and find the temperature of the entire system by fitting the kinetic energy distribution to a Maxwell–Boltzmann distribution, defined by a temperature.

We perform simulations up to carrier densities of 10^19^ cm^–3^. Here, particle statistics are no longer accurately described by the classical Maxwell–Boltzmann regime, as one now enters the quantum mechanical Fermi–Dirac regime.^66^ Our main interest lies in high-energy (high temperature) particle dynamics. At reasonably high temperatures, there are vastly more energy states than particles. Therefore, the majority of states are empty, and the probability of two particles attempting to occupy the same state is negligible. In this limit, the denominator of the Fermi–Dirac statistics approaches exp(−*E*/*k*_*b*_*T*), which is recognized as the Maxwell–Boltzmann distribution.^67^ The use of Maxwell–Boltzmann statistics is therefore still appropriate.

For the implementation of the HPB effect, the EMC was extended with the algorithm developed by Lugli et al.^51^ For a full description of the algorithm we suggest the original literature.^51,52,68^ We present a summary in the Supporting Information and provide the essential equations below.

The dynamic evolution of the LO phonon population is described by the following difference equation:^68^
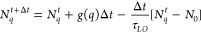
3where *g*(*q*) is given by
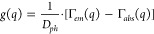
4with Γ_*em*_(*q*) the LO phonon emission probability, and Γ_*abs*_(*q*) the LO phonon absorption probability, and *D*_*ph*_ the phonon density of states.

The phonon density of states is given by
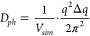
5with *V*_*sim*_ the simulation volume, *q* the phonon wavevector, and Δ*q* the cell size of the q-space histogram. Here, we factor in the simulation volume, and use the general expression for the phonon density of states for a symmetric perfect crystal.^69^ Here, Δ*q* is a free parameter and was taken to be 10^6^ cm^–1^. Choosing Δ*q* is a matter of achieving good statistics.^70^ One should not pick Δ*q* to be too small, as now the perturbation of the phonon population will not properly be captured.

During the entirety of the simulation time, one continuously solves eq 3, and updates the scattering rates via eq 2 accordingly, with updated values for *N*_*q*_.

We commence with the concentration dependence of the HPB effect in Figure 2, for particle densities between ρ = 10^16^ cm^–3^ and ρ = 10^19^ cm^–3^. Here, the parameters of the perovskite system were given by m* = 0.2, ε_0_ = 30, ε_*∞*_ = 10, and ω_0_ = 50 ps^–1^. This system is comparable with a MAPbI_3_ system regarding its effective mass and static dielectric constant ε_0_, with ε_*∞*_ and ω_0_ belonging to a lighter halide system. In these simulations, we kept the lifetime of the phonons constant at τ_*LO*_ = 0.1 ps.

**Figure 2:**
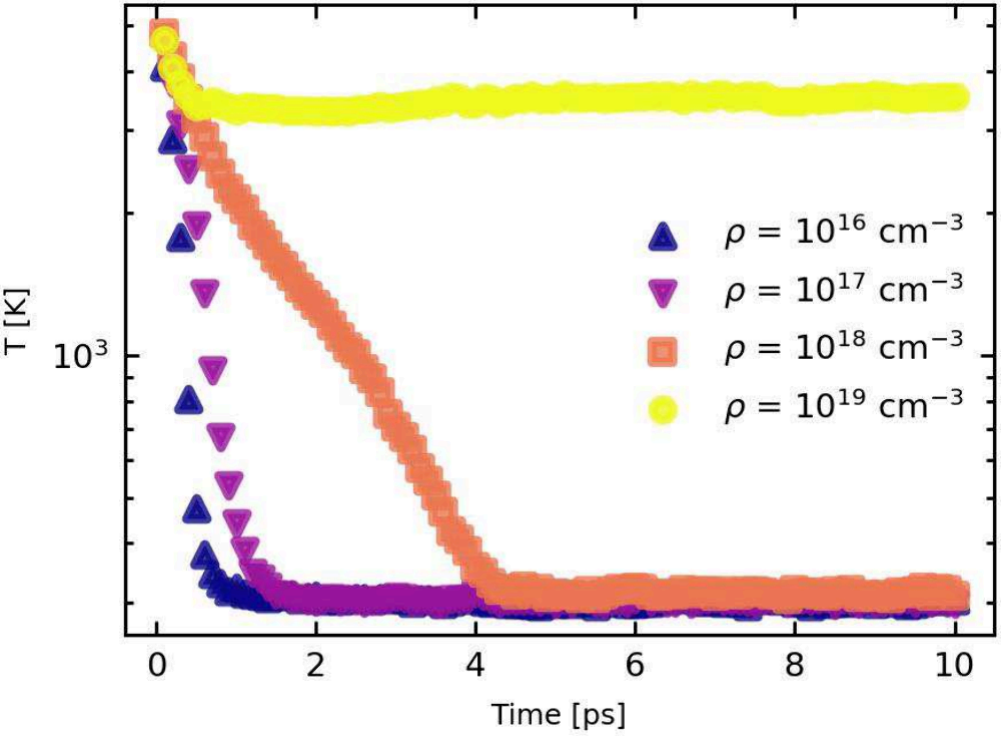
Carrier cooling times for different particle densities ranging from ρ = 10^16^ cm^–3^ to ρ = 10^19^ cm^–3^. T corresponds the carrier temperature.

We observe that cooling is impacted at a carrier concentration of 10^18^ cm^–3^, matching what was reported experimentally for when the HPB would start playing a role in MHP systems.^4^ Going up to higher carrier densities (ρ = 10^19^ cm^–3^), we observe a dramatic increase in the cooling time.

In Figure 3 we plot the ratio of the absorption over the emission scattering rate Γ_*abs*_/Γ_*em*_. The HPB effect will increase the phonon occupation number to values above its equilibrium value, bringing the emission and absorption scattering rates closer together (See eq 2).

**Figure 3:**
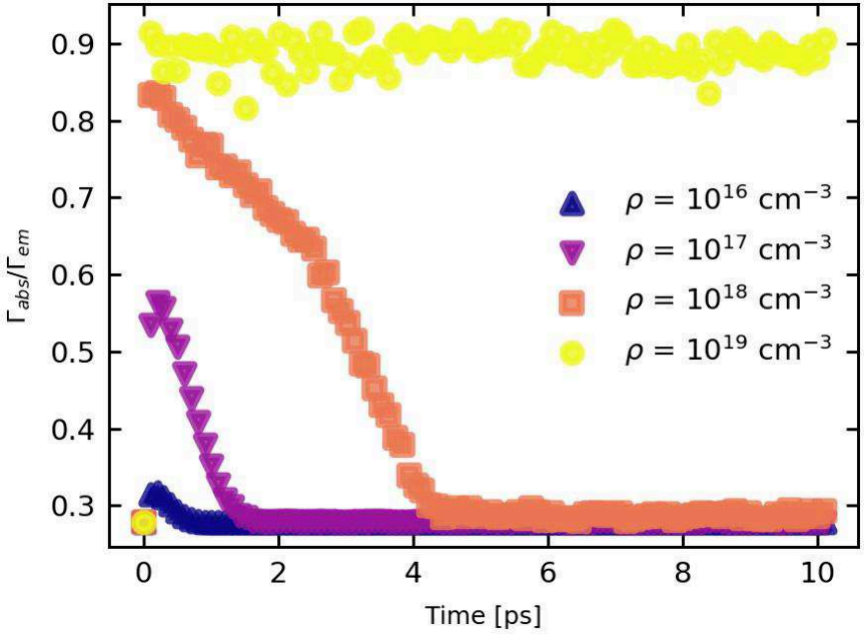
Ratio of the absorption and emission scattering rate for different particle densities ranging from ρ = 10^16^ cm^–3^ to ρ = 10^19^ cm^–3^.

At a high particle density (ρ = 10^19^ cm^–3^), Γ_*abs*_/Γ_*em*_ is very close to unity, and remains so for the entirety of the simulation time. A significant number of phonons are generated, severely hindering the system’s cooling process. This stretches the cooling time by orders of magnitude, well beyond the LO phonon lifetime τ_*op*_. The carrier reabsorption rate is in an absolute sense much larger than the optical phonon decay rate. LO phonons are reabsorbed before they have a chance to decay.

By fitting an exponential to the data of Figure 2, we can obtain a measure for the cooling time for different particle densities, provided in Figure 4. We observe a strong nonlinear increase, illustrating the effect of an increasing reabsorption rate.

**Figure 4:**
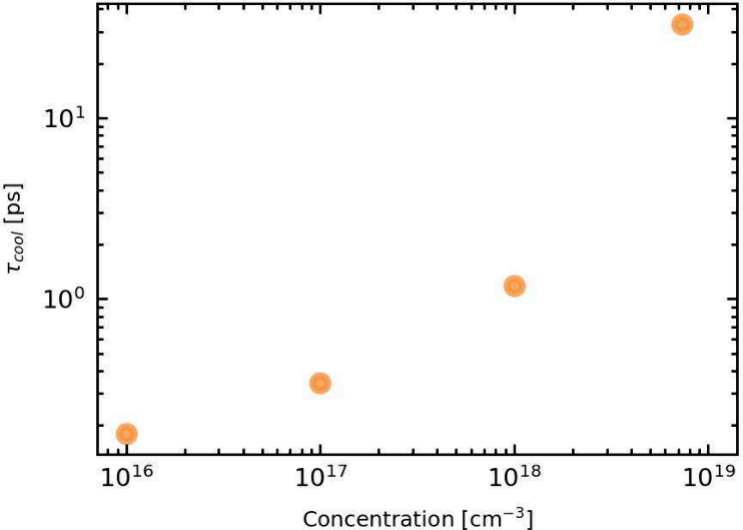
Cooling time vs concentration for ρ = 10^16^ cm^–3^ up to ρ = 10^19^ cm^–3^ with τ_*op*_ = 0.1 ps. We observe a nonlinear increase in the cooling time with increasing particle density.

What is clear from these results is that a small variation in carrier density will yield significantly different outcomes for the carrier cooling time. In their central study regarding the hot phonon bottleneck in perovskites, Yang et al. show for MAPbI_3_, an increase in carrier density from ρ = 1.5 × 10^18^ cm^–3^ to ρ = 6 × 10^18^ cm^–3^, corresponds to more than an order of magnitude increase in the cooling time from around 3 ps to approximately 80 ps.^6^ This corresponds quite well with our findings, and highlights the potential of the HPB effect.

This point is not only an important statement regarding the potential for future HCSC applications, but also of relevance in explaining the variation in experimentally measured cooling times. Only a small uncertainty in the reported pump fluence would result in very different carrier cooling times.

In Figure 5 the dependence of the HPB effect on τ_*LO*_ is provided. The simulations were performed on a similar perovskite-like system (m* = 0.15, ε_0_ = 30, ε_*∞*_ = 10, and ω_0_ = 31 ps^–1^), at a particle density of ρ = 10^18^ cm^–3^. Cooling times start extending between τ_*LO*_ = 0.1 and 1 ps, comparable with experimental values found for perovskites.^7,43^ For even longer LO lifetimes τ_*LO*_, one can observe that we can elongate the cooling times significantly.

**Figure 5:**
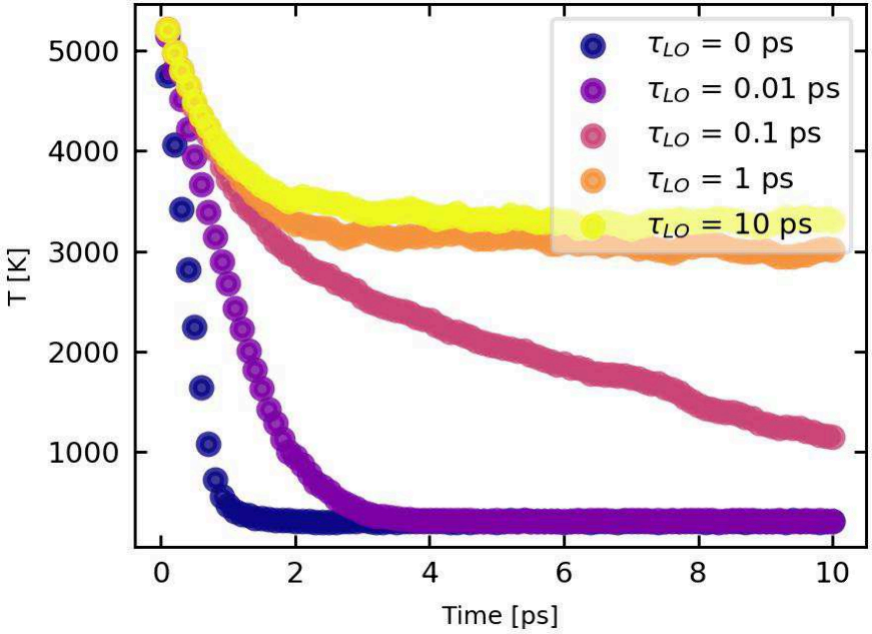
Carrier cooling times for different values of the phonon lifetime τ_*LO*_ ranging from 0 to 10 ps at a carrier density of ρ = 10^18^ cm^–3^.

Phonon lifetimes can be impacted by altering the halide composition of the perovskite.^71^ However, by comparing three Pb compounds, Leguy et al. show that a change of halide does not so much alter the optical modes, which couple most strongly to the carriers.^71^ The authors do point out that increased coupling between the organic and inorganic sublattices leads to a broadening of the Raman peaks.^71^ This broadening, characterized by the full-width half-maximum, is inversely proportional to the associated phonon lifetime. Therefore, one could attempt to limit the interaction between the organic and inorganic sublattices. This could be achieved for example by the substitution of an inorganic A-site cation.^72,73^

Another way to increase the phonon lifetime is by finding a material with a phononic band gap.^74^ Reports have found evidence of a phononic bandgap in lead-based perovskites, suppressing the Klemens decay, as a main decay channel for LO phonons.^7,75^ A phononic band gap arises from the large difference between the anion and cation masses.^36,76^ This could be further explored by the substitution of a heavier B-site cation,^77^ or lighter halide.^78^ An increase of τ_*LO*_ to only a few ps, would have very significant effects on the carrier cooling time.

Finally, also up-conversion of other types of phonons back to LO phonons (Reversal of stage 2 in the introduction) must be noted. Up-conversion of acoustic phonons was already mentioned by Yang et al.,^36^ and more recently by Sekiguchi et al., via excitation of TO phonons.^10^ Strong phonon–phonon interactions arise due to a high degree of anharmonicity, originating from the soft Pb–I cage.^10^ This results in very short acoustic phonon lifetimes, and therefore a higher probability for up-conversion.^36,79,80^ A further exploration of materials with ultralow thermal conductivity is therefore worthwhile.

Finally, we have investigated the role of the typical phonon frequency ω_0_, shown in Figure 6, for the same perovskite-like system (m* = 0.15, ε_0_ = 30, ε_*∞*_ = 10) at ρ = 10^18^ cm^–3^, with τ_*LO*_ = 0.1 ps.

**Figure 6:**
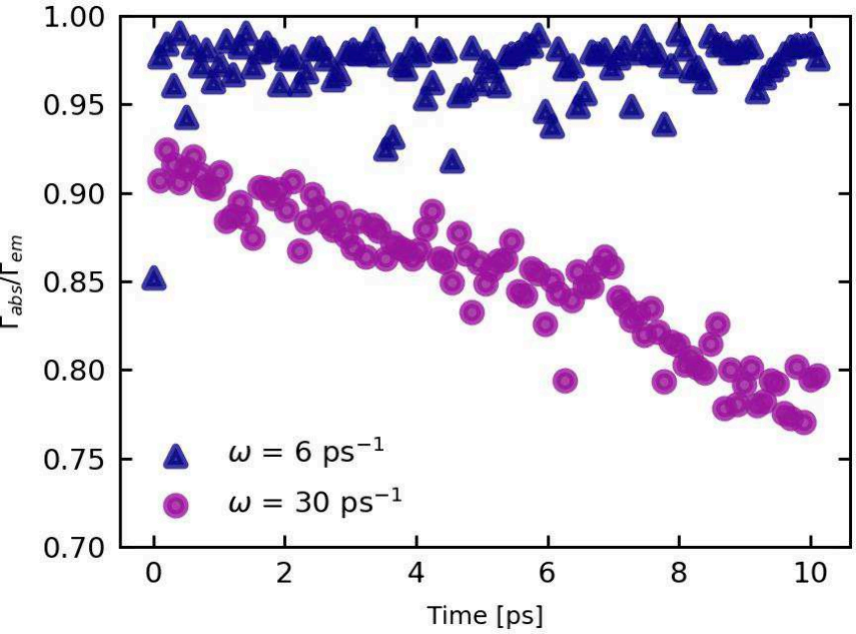
Ratio of the absorption and the emission scattering rate for different typical phonon frequencies ω_0_ = 6 ps^–1^ and ω_0_ = 30 ps^–1^.

From Figure 6, we observe that for a smaller frequency ω_0_ = 6 ps^–1^, the ratio Γ_*abs*_/Γ_*em*_ edges much closer to unity. The HPB effect is shown to be more prominent in lower frequency systems.

The role of the typical phonon frequency ω_0_ is not entirely trivial. The quantity returns in the carrier–phonon scattering rate twice. Directly via ∼ ω_0_ (see eq 2): As the LO phonon energy is smaller, more phonons must be emitted before carriers end up in their respective band edges. However, also in a more subtle, indirect way, via the phonon occupation number *N*_*q*_. After reaching the peak, the phonon occupation number will relax back to its equilibrium value, which is given by
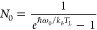
6This quantity will be large, for a smaller value of ω_0_, and thus the ratio Γ_*abs*_/Γ_*em*_ will be smaller in equilibrium. Hence, carrier cooling is in general less efficient for systems with smaller values of ω_0_. Additionally, we show here that the HPB effect will be more effective for systems with a smaller typical phonon frequency ω_0_. It is therefore highly fruitful to have a material possessing a small LO-phonon frequency. A possible way to reach smaller LO phonon energies is by switching from a lighter (Cl^–^) to a heavier (I^–^) halide.^74,81^

Our study shows how one can significantly extend carrier cooling times by a combined optimization of the carrier concentration, LO phonon lifetime, and LO phonon frequency. Regarding HCSCs care should be taken. On one hand, the need for high carrier concentrations bodes well with the necessity of requiring fast thermalisation to repopulate the extraction levels.^82^ On the other hand, this would raise serious issues with material integrity and device stability.^78^

Finally, at high carrier densities, Auger recombination will also start playing a role.^7,83^ In order to capture the potential of the HPB effect, without experiencing too much interference of Auger recombination, no extreme carrier densities should be used. Our results show that already at densities around ρ ∼ 10^18^, a significant boost in cooling time could be obtained by the HPB effect.

We present an investigation of the hot phonon bottleneck (HPB) effect in metal halide perovskites using ensemble Monte Carlo simulations. We have shown, by studying the carrier concentration, LO-phonon lifetime, and LO-phonon frequency, how the HPB effect can extend cooling times by orders of magnitude, far beyond the LO phonon lifetime. Our study connects these findings to concrete chemical compositions for metal halide perovskites, and shows that the HPB effect could play a key role for future HCSC applications.

## Supporting Information Available

Extended overview of EMC and hot phonon bottleneck methodology (PDF)
