# Supplementary material for: The Hot Phonon Bottleneck Effect in Metal Halide Perovskites
Source: J Phys Chem Lett. 2024 Dec 16;15(51):12601–7. doi: 10.1021/acs.jpclett.4c03133 (PMC11684017; doi:10.1021/acs.jpclett.4c03133)
Supplement: Supplementary file 1 — jz4c03133_si_001.pdf [file jz4c03133_si_001.pdf]

Supporting Information: The hot phonon bottleneck effect in metal halide perovskites

T. Faber,† L. Filipovic,‡ and L.J.A. Koster†

†*Zernike Institute for Advanced Materials, University of Groningen, Nijenborgh 4, 9747 AG Groningen, The Netherlands.*

‡*CDL for Multi-Scale Process Modeling of Semiconductor Devices and Sensors at the Institute for Microelectronics, TU Wien, Gusshausstrasse 27-29 1040 Vienna, Austria.*

Email: l.j.a.koster@rug.nl

Ensemble Monte Carlo (EMC)

In the EMC method randomly generated free flights are interrupted by randomly selected scattering events,[1] translating the chaotic motion of the thermal ensemble using stochastic methods. The EMC method is semi-classical. The free flight is modeled classically, while the scattering rates are evaluated quantum mechanically via Fermi's Golden Rule.[2] The scattering rates are stored in intervals of 0.4 meV look-up tables, which are energy-dependent and can be pre-computed. As in our previous work,[3] our model consists of carrier-phonon interactions and carrier-carrier interactions, incorporating all fundamental processes governing the cooling dynamics in MHPs.[4] The LO-phonon interactions are described by Fröhlich interactions,[5] and carrier-carrier interactions via Coulomb's law.

For the carrier-carrier interactions, a $(r + a)$ kernel was used, with a cut-off radius of $a = 1$ nm. A cut-off radius ensures numerical stability using A fast multipole method (FMM), while staying well below the average interparticle distance.[6,7] The Coulomb potential is then given by

$$V_c(r) = \frac{q}{4\pi\epsilon(r + a)}. \tag{1}$$

Here, we set ϵ equal to the optical part of the dielectric constant ϵ_∞. Our potential was solved up to second order, yielding a maximum computation error of 10^{-2}. Ensembles of 10000 to 100000 particles were simulated in a 100nm to 1 µm box, which was periodically repeated in all three directions 384 times. Particle ensembles consisted of 50% holes and 50% electrons. We used a parabolic band structure approximation described by the effective mass. For a more extensive description we direct the reader to our previous paper,[3] and the original paper of Jacoboni et al.[2,8]

Hot phonon bottleneck in EMC simulations

The physical quantity that determines the amplitude of the HPB is the LO phonon population N_q. The dynamic evolution of the LO phonon population can be described by[9]

$$\frac{\partial N_q}{\partial t} = \left.\frac{\partial N_q}{\partial t}\right|_{c-ph} - \left.\frac{\partial N_q}{\partial t}\right|_{ph-ph}. \tag{2}$$

The first term on the right, the carrier-phonon contribution, could be solved directly by the implementation of Fermi's Golden Rule.[10] Fu et al. have done so, yielding some fruitful insights explaining extended cooling times with the HPB effect in $MAPbI_3$.[11] However as we aim to give a complete overview of the entire cooling dynamics in MHPs, incorporating carrier-carrier interactions in the process, we solve this term using the EMC.

The second term on the right can be approximated using a single-mode

relaxation time approximation

$$\left.\frac{\partial N_q}{\partial t}\right|_{ph-ph} = \frac{N_q - N_0}{\tau_{LO}}, \tag{3}$$

with $N_0 = 1 \;/\; \exp(\hbar\omega_0/k_b T_L)$, the equilibrium phonon occupation at the lattice temperature T_L given in equilibrium by the thermal Planck distribution, equal for all phonons with different momenta q. τ_{LO} is the LO-phonon lifetime. A general lifetime τ_{LO} is taken for all values of q, the phonon momentum. This is not a necessary approximation to make; however, a reasonable and convenient one. We take this parameter as a free parameter in our model to investigate its effect.

As a result of using numerical methods (i.e., EMC), one must continue rewriting (2) into a difference equation. Together with the insertion of (3), the value of N_q at timestep $t + \Delta t$ is given by

$$N_q^{t+\Delta t} = N_q^t + g(q)\Delta t - \frac{\Delta t}{\tau_{LO}}[N_q^t - N_0], \tag{4}$$

where $g(q)$ is given by

$$g(q) = \frac{1}{D_{ph}} \cdot [\Gamma_{em}(q) - \Gamma_{abs}(q)], \tag{5}$$

with $\Gamma_{em}(q)$ the LO phonon emission probability, $\Gamma_{abs}(q)$ the LO phonon absorption probability, and D_{ph} the phonon density of states.

The term $g(q)$ yields, for every value of q, the difference between the number of phonon emission events and phonon absorption events. More concretely, it tracks how many phonons are produced/lost via carrier-phonon interactions at each timestep. As these events can be marked within the EMC, one can obtain a full phonon distribution for each timestep.

One must keep track of the entire population, as the phonon occupation number N_q is not equal for each value of q when the system is outside of equilibrium. This can be seen from Eq. 1, where we note the scattering amplitude falling off as q^{-2}, resulting in a non-equilibrium population characterized by the 'hot' phonons near $q > 0$.

The phonon population is tracked by making use of a discretization in q-space. Only the amplitude of q is required, therefore a 1-dimensional vector suffices. For every scattering event with a LO phonon, the value for q is determined, and the appropriate cell in q-space is decremented in the case of an emission event, or incremented in the case of an absorption event.[12]

The phonon density of states is given by

$$D_{ph} = \frac{1}{V_{sim}} \cdot \frac{q^2 \Delta q}{2\pi^2} \tag{6}$$

with V_{sim} the simulation volume, q the phonon wavevector, and Δq is the cell size of the q-space histogram. Here, we factor in the simulation volume, and use

the general expression for the phonon density of states for a symmetric perfect crystal.[13] Here, Δq is a free parameter to choose from and was taken to be 10^6 cm^{-1}. Choosing Δq is a matter of achieving good statistics.[14] One should not choose Δq too small, as now the perturbation of the phonon population will not properly be captured.

References

[1] Karel Nederveen. *Ensemble Monte Carlo simulation of electron transport in AlGaAs/GaAs heterostructures.* PhD thesis, Electrical Engineering, 1989.

[2] C. Jacoboni and P. Lugli. *The Monte Carlo Method for Semiconductor Device Simulation.* Computational Microelectronics. Springer Vienna, USA, 2011.

[3] Tim Faber, Lado Filipovic, and L. Jan Anton Koster. The role of thermalization in the cooling dynamics of hot carrier solar cells. *Solar RRL*, 7(13):2300140, 2023.

[4] Thomas R Hopper, Andrei Gorodetsky, Jarvist M Frost, Christian Muller, Robert Lovrincic, and Artem A Bakulin. Ultrafast intraband spectroscopy of hot-carrier cooling in lead-halide perovskites. *ACS energy letters*, 3(9):2199–2205, 2018.

[5] H. Fröhlich and N. F. Mott. Theory of electrical breakdown in ionic crystals. ii. *Proceedings of the Royal Society of London. Series A. Mathematical and Physical Sciences*, 172(948):94–106, 1939.

[6] C. Heitzinger, Christian Ringhofer, Shaikh Ahmed, and D. Vasileska. Efficient simulation of the full coulomb interaction in three dimensions. *Journal of Computational Electronics*, 11:24, 2004.

[7] Laura Gollner. *Development and Application of an Ensemble Monte Carlo Framework.* MSc Thesis, TU Wien, Vienna, Austria, 2023.

[8] C Jacoboni and L Reggiani. The monte carlo method for the solution of charge transport in semiconductors with applications to covalent materials. *Reviews of Modern Physics*, 55:645–705, Jul 1983.

[9] Paolo Lugli. Hot phonon dynamics. *Solid-State Electronics*, 31(3):667–672, 1988.

[10] M. Pugnet, J. Collet, and A. Cornet. Cooling of hot electron-hole plasmas in the presence of screened electron-phonon interactions. *Solid State Communications*, 38(6):531–536, 1981.

[11] Jianhui Fu, Qiang Xu, Guifang Han, Bo Wu, Cheng Hon Alfred Huan, Meng Lee Leek, and Tze Chien Sum. Hot carrier cooling mechanisms in halide perovskites. *Nature communications*, 8(1):1300, 2017.

[12] David K Ferry. The ensemble monte carlo method. In *Hot Carriers in Semiconductors*, 2053-2563, pages 7–1 to 7–49. IOP Publishing, USA, 2021.

[13] C. Kittel and P. McEuen. *Introduction to Solid State Physics.* Wiley, 2018.

[14] David Ferry. *Semiconductor transport.* CRC Press, 2016.
